# Lithium management of periodic mood fluctuations in behavioural frontotemporal dementia: a case report

**DOI:** 10.3389/fpsyt.2023.1325145

**Published:** 2024-01-08

**Authors:** Vicent Llorca-Bofí, Iolanda Batalla, Maria Ruiz-Julián, Marina Adrados-Pérez, Esther Buil-Reiné, Gerard Piñol-Ripoll, Xavier Gallart-Palau, Aurora Torrent

**Affiliations:** ^1^Department of Medicine, University of Barcelona School of Medicine, Barcelona, Spain; ^2^Department of Psychiatry, Hospital Universitari Santa María, Lleida, Spain; ^3^Institut de Recerca Biomèdica de Lleida (IRBLleida), Lleida, Spain; ^4^Medicine Department, Universitat de Lleida (UdL), Lleida, Spain; ^5^Cognitive Disorders Unit, Hospital Universitari Santa Maria, Lleida, Spain; ^6^Biomedical Research Institute of Lleida Dr. Pifarré Foundation (IRB Lleida), Neuroscience Area, +Pec Proteomics Research Group (+PPRG), University Hospital Arnau de Vilanova (HUAV), Lleida, Spain; ^7^Psychology Department, University of Lleida (UdL), Lleida, Spain; ^8^Faculty of Health Sciences, Valencian International University, Valencia, Spain

**Keywords:** behavioural frontotemporal dementia, mood fluctuations, manic-like episodes, lithium, case report

## Abstract

The behavioural variant of Frontotemporal Dementia (bvFTD) is a neurodegenerative condition characterized by behavioural and cognitive symptoms. Mood disturbances, including manic-like episodes, can occur in bvFTD, posing diagnostic and therapeutic challenges. This case report presents a 62-year-old male with bvFTD exhibiting weekly mood fluctuations alternating between manic and depressive-like states. While initial treatment with quetiapine and trazodone showed partial improvement, the periodicity of mood fluctuations persisted. Subsequently, lithium was introduced, resulting in a notable reduction in symptom severity for both manic and depressive episodes. This report highlights the potential use of lithium as a mood stabilizer in bvFTD patients with periodic mood fluctuations, refractory to standard treatments. Further research is needed to elucidate the mechanisms underlying lithium’s efficacy in bvFTD and to establish treatment guidelines.

## 1 Introduction

Frontotemporal Dementia is the third leading cause of neurodegenerative dementia, with the most common form being the behavioural variant (bvFTD) (1). Initial mood and personality disturbances are a characteristic feature of bvFTD, often manifesting as a loss of empathy, apathy or abulia, and disinhibition or impulsivity. However, bvFTD presents a wide range of behavioural symptoms, which is why many patients are eventually referred to the Psychiatry service (2). In this setting, the differential diagnosis between bvFTD and a primary psychiatric disease (3) is stablished, and if needed, appropriate adjustments are made to psychopharmacological interventions.

Among the initial behavioural changes, patients may present with euphoria, increased energy, loquacity and racing thoughts, irritability, distractibility, and disinhibition. This presentation resembles a manic or hypomanic episode and there are numerous reports of manic behaviour as an initial manifestation of bvFTD (4). However, the occurrence of periodic mood fluctuations resembling bipolar disorder in bvFTD is relatively rare. Additionally, the use of lithium for managing bvFTD has been infrequent and is currently under study (5, 6). This case report describes a patient with bvFTD who experienced weekly mood fluctuations consisting of manic and depressive-like symptoms. Subsequently, lithium was introduced as a treatment option after a partial response to conventional therapy.

## 2 Case description

We present the case of a 62-year-old man who was evaluated by the Neurology service for functional decline and progressive behavioural alteration. The patient had a tobacco use disorder but did not exhibit any other toxic habits. He had a history of successfully treated chronic hepatitis caused by hepatitis C virus and no prior psychiatric history. Apart from a first-degree relative who experienced a stroke in old age, there was no other family history of neurological diseases, including dementia, or psychiatric disorders.

**Figure 1:**
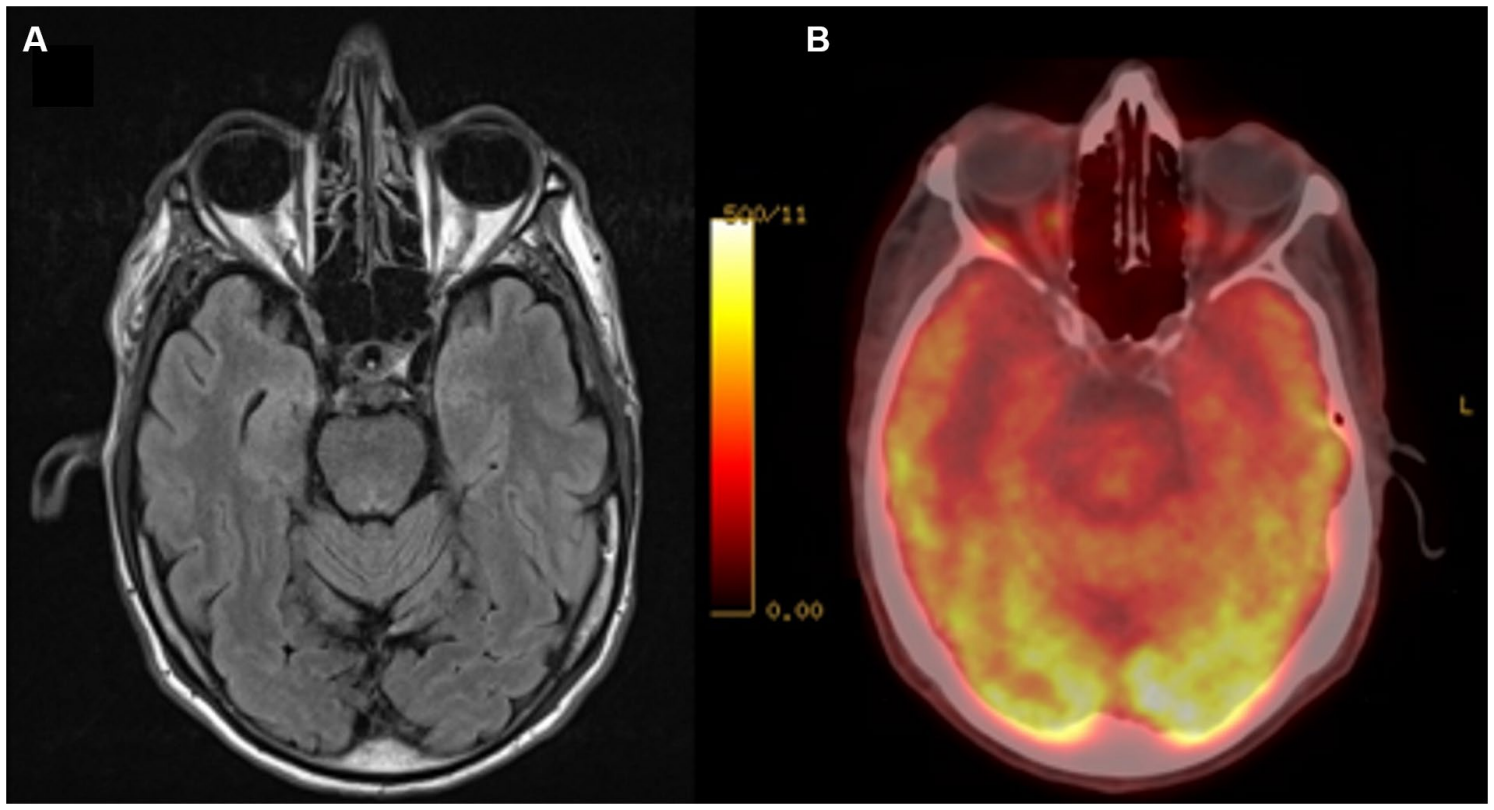
Neuroimaging Exams. **(A)** Brain MRI, FLAIR sequence; **(B)** Brain PET/CT with 18F-FDG. A decrease in glycidic metabolism is observed at the bilateral anterior temporal level.

At the neurologic assessment, his spouse explained an insidious onset of behavioural changes approximately three years earlier, which ranged from irritability to apathy and anergy in the same week with progressive deterioration of functionality at home. The patient lacked insight and relativized his functional deficits. In the initial evaluation, no alterations were observed in the screening tests, including MMSE, clock test, praxis, Luria motor sequence and graphic alternations. A blood test with cobalamin, folic acid, TSH and luetic serology was performed with normal results. A brain MRI did not show atrophy or other significant findings. Glucose PET showed mild hypometabolism at the bilateral anterior temporal level, with preservation of metabolism in the rest of the cortical and subcortical structures (Figure 1). The neuropsychological examination revealed a deficit in executive functions (cognitive flexibility and alternation) and low performance in immediate visual memory, with normality in the rest of the cognitive areas evaluated. Quetiapine 25 mg was started at night, yielding limited efficacy, most likely attributable to suboptimal adherence resulting from the reported sensation of weakness. Subsequently, the patient was referred to the Psychiatry service for comprehensive assessment of the observed behavioural alteration.

On psychiatric assessment, the wife explained a fluctuant behavioural pattern with a weekly frequency. During manic-like episodes, the patient was particularly happy and full of energy, but would become irritable if contradicted in any way. They spent most of the day in a heightened state, rearranging furniture at home and making unrealistic plans. They repeatedly confronted the neighbours, displaying uninhibited behaviour, and slept only a few hours, claiming to have enough energy to not sleep. In the psychopathological examination the patient was awake, conscious and oriented in all 3 spheres. He was approachable and collaborative and at times presented loss of social distance. The patient appeared partially well-groomed, with some internal tension and the contact was syntonic. The speech was accelerated and under pressure, circumstantial and sometimes disorganised, with a loss of purpose and with humorous content standing out. The affect was expansive with hyperthymia. He did not present suicidal ideation. He also had no psychotic symptoms or altered sensory perception. He highlighted basal anxiety with irritability. Sleep decreased to 2–3 h despite quetiapine treatment. No self or hetero-aggressive behaviour was shown. Insight was null with a marked lack of empathy. During this period the clinical scores were YMRS (7): 33 and MADRS (8): 9.

In the following 4–5 days, the patient remained calm, correct and adequate, resting all night but listless, sad and lacking in energy. He presented pessimistic thoughts of guilt but without suicidal ideation. In the subsequent psychopathological examination, the patient was approachable and cooperative, well-groomed and did not present alterations in psychomotricity. The contact was syntonic. His speech was limited, concrete and impoverished with little fluency and without reaching objectives. Affect was flattened and he presented hypothymia with negative cognitions, apathy, abulia, and hypohedonia. He did not present suicidal ideation. He also had no psychotic symptoms or altered sensory perception. During this period there was no anxiety or irritability and sleep was pharmacologically corrected. No self or hetero-aggressive behaviour was shown. The insight was null. Clinical scores were YMRS: 7 and MADRS: 22. Throughout these periods, he presented difficulties in planning his day-to-day activities, which was accentuated as the week went by until the start of the 2–3 mania-like days and the cycle started again.

A strict application of the DSM-5 criteria (9) for bipolar disorder ruled out this diagnosis. The patient did not meet criterion A (showed less than one week of manic symptoms) for a manic episode. He also did not meet criterion A (showed less than two weeks of depressive symptoms) for a major depressive episode. He did not meet criterion B (he showed more than two consecutive months with symptoms present) for cyclothymic disorder. He also did not meet the criteria for rapid cycling bipolar disorder because he did not meet the definitions of mania or major depressive disorder. Some authors have proposed the term “ultra-rapid cycling” when cycles occur with a frequency of days to weeks (10). Although it is not included in the DSM-5 specifiers, this presentation could bear resemblance to that of our patient if not for the pronounced presence of neurocognitive disturbances.

Regarding the potential classification as a mixed episode, the patient did not concurrently manifest symptoms of both polarities. Instead, distinct periods of manic or depressive-like symptoms were evident. Furthermore, the patient did not satisfy criterion A (failed to meet all the criteria for a depressive or manic episode) for a mixed episode. Concerning bipolar disorder due to another medical condition, the development of neurocognitive difficulties oriented more toward a dementia-type process and therefore excluded criterion C (better explained by another medical condition) for this diagnosis. He was diagnosed with periodic mood fluctuations in a probable behavioural frontotemporal dementia based on the 2011 Rascovsky diagnostic criteria (11). Following current recommendations (3), C9orf72 mutation was studied, which was negative.

Based on the results of the clinical trial by Lebert et al. (12), trazodone was started in ascending doses up to 300 mg at night with good tolerance and quetiapine was stopped. Mania-like episodes were reduced in duration (from three to one day) and severity (YMRS: 26) and depressive-like episodes were reduced in severity (MADRS: 16), but the fluctuations of the mood periods remained weekly and insight remained impaired.

**Figure 2:**
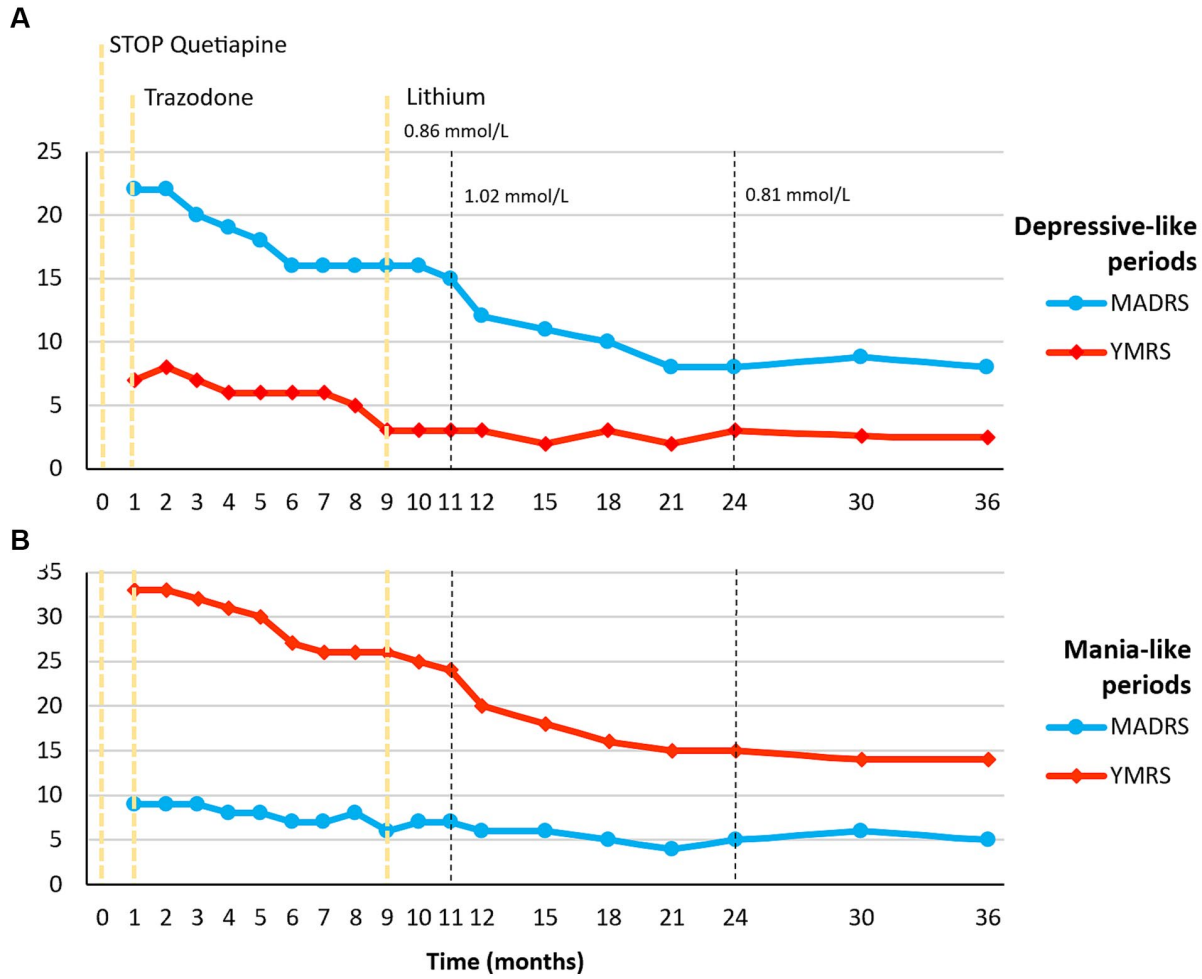
Timeline of Pharmacological and Psychopathological Changes. **(A)** Psychopathological changes measured by MADRS and YMRS in depressive-like periods; **(B)** Psychopathological changes measured by MADRS and YMRS in mania-like periods. An initial improvement in both mania and depressive-like symptoms was observed during trazodone treatment, reaching a plateau in the response at month 9. Subsequently, the addition of lithium resulted in a greater improvement, and this response was sustained for up to 3 years of follow-up. MADRS, Montgomery-Asberg Depression Rating Scale; YMRS, Young Mania Rating Scale.

After six months of treatment with trazodone at the maximum dose, we introduced lithium to leverage its mood-stabilizing effects. We started with a nightly dose of 200 mg, gradually increasing it by 200 mg every seven days, reaching a daily dose of 600 mg within one month. Despite achieving blood lithium levels of 0.86 mmol/L in the subsequent month (see Figure 2, month 10), minimal effects on both mania and depression were observed (YMRS 25 to 24; MADRS 16 to 15), and the weekly cyclicity persisted.

During the next visit (see Figure 2, month 11), following the recommendations for managing acute mania (13), we adjusted the dosage to 800 mg/day, resulting in a blood lithium level of 1.02 mmol/L. Over the following ten months, a significant reduction in symptom intensity occurred during both manic and depressive phases, yielding scores of YMRS: 15 and MADRS: 8 at month 24, albeit with ongoing weekly cyclicity.

Subsequently, following the current recommendations for maintenance treatment (14), we reverted to a dose of 600 mg/day with blood lithium levels stabilizing at 0.81 mmol/L. We continued monitoring the patient up to 36 months since the initial contact, with no significant changes in clinical severity observed. Throughout both the acute episode and the follow-up, the patient reported no relevant adverse effects. Kidney and thyroid function remained within normal ranges, and calcium levels were also normal.

## 3 Discussion

Cognitive and behavioural symptoms are the hallmark of bvFTD (1). However, mood disturbances, such as depression, and less frequently, manic episodes, can also manifest in this condition (2). Managing mood fluctuations in neurological disorders presents significant challenges, as it deviates from the established diagnostic criteria for bipolar disorder or other psychiatric conditions (15, 16). This case report provides evidence of the potential effectiveness of lithium in treating periodic mood fluctuations in bvFTD.

The management of mood disturbances in bvFTD is complex and often requires a multimodal approach. Pharmacological interventions for mood disturbances in bvFTD have primarily focused on selectively targeting the serotonergic system affected in FTD (15). Trazodone, at doses of at least 300 mg/day, has shown both acute and long-term benefits in managing behavioural alterations in bvFTD (12, 17). Lithium has also been proposed as a potential treatment for manic symptoms in bvFTD (18). However, the existing evidence comes from case reports describing favourable outcomes with the use of lithium in the treatment of mood symptoms (5). Nonetheless, a phase II randomized clinical trial is currently underway to evaluate the efficacy of low-dose lithium on behavioural symptoms in patients with bvFTD, which will provide valuable evidence in this field (6).

In the present case, the patient exhibited periodic mood fluctuations with weekly periodicity, alternating between manic-like and depressive-like periods. Initial treatment with quetiapine and trazodone provided partial relief, but the weekly cyclicity of mood fluctuations persisted. To address the mood instability observed in the patient, lithium was added to the treatment regimen. Notably, the addition of lithium with serum until 1.2 mmol/L resulted in a significant reduction in the intensity of both manic and depressive symptoms. In a published case series of three patients with bvFTD or semantic variant-primary progressive aphasia, lithium used at serum concentrations ranged between 0.4 and 0.8 mmol/L daily improved behavioural disturbances notably agitation with or without psychotic features (5). However, in our approach, higher doses of lithium were used in the acute episode, with a focus on mood disturbances rather than behavioural symptoms. These clinical findings support the consideration of lithium as a potential therapeutic option for managing mood fluctuations in bvFTD that have not shown a response to the currently recommended treatments (19).

Lithium is a well-established mood stabilizer primarily utilized in the treatment of bipolar disorders (20). Its therapeutic effects stem from diverse mechanisms, including the modulation of intracellular signaling pathways, neuroprotective actions, and regulation of neurotransmitter systems (21). In the context of bvFTD, the mood-stabilizing effects of lithium are believed to result from its capacity to modulate intracellular signaling pathways (20, 21). Specifically, lithium exhibits inhibitory effects on glycogen synthase kinase-3β (GSK-3β), a factor involved in tau phosphorylation. By influencing abnormal protein aggregation, lithium is hypothesized to act as a neuroprotective agent against tauopathies, including bvFTD. However, the precise mechanism of action for treating mood disturbances in bvFTD remains speculative. Translational research suggests neurotrophic and neuroprotective effects that contribute to the modulation of multiple homeostatic mechanisms, which are core pathological processes in dementia (22). Additional research is necessary to clarify the specific mechanisms through which lithium operates in bvFTD.

It is important to note that the response to lithium in bvFTD-associated mood fluctuations can be variable, and not all patients may experience significant benefit. In our case report, the addition of lithium to the treatment regimen resulted in a reduction in symptom severity (Figure 2), but the weekly cyclicity of mood fluctuations persisted. Insight remained impaired, indicating that lithium may primarily target mood symptoms rather than underlying cognitive deficits. However, we recognize the limited generalizability inherent in single-case studies. To address this, we advocate for further research with larger samples. The ongoing randomized clinical trial (6) will surely provide valuable information on the acute effects, but longitudinal studies will also be necessary to investigate the sustained efficacy of the intervention. These efforts will contribute to a more robust evidence base and guide the development of targeted treatment guidelines for this challenging condition.

In conclusion, this case report provides evidence for the potential use of lithium as a mood stabilizer in the management of periodic mood fluctuations in bvFTD after an unsatisfactory response to conventional treatments. While the exact mechanisms underlying the therapeutic effects of lithium in bvFTD are not fully understood, its neuroprotective properties and modulation of neurotransmitter systems may play a role. Further research is needed to validate these findings and to guide the use of lithium in bvFTD.

## Data availability statement

The raw data supporting the conclusions of this article will be made available by the authors, without undue reservation.

## Ethics statement

The studies involving humans were approved by Arnau de Vilanova University Hospital – GSS, Lleida, Spain (CEIC-2341). The studies were conducted in accordance with the local legislation and institutional requirements. The participant provided his written informed consent to participate in this study. Written informed consent was obtained from the individual for the publication of any potentially identifiable images or data included in this article.

## Author contributions

VL-B: Writing – original draft, Writing – review & editing. IB: Writing – original draft, Writing – review & editing. MR-J: Data curation, Formal analysis, Investigation, Writing – review & editing. MA-P: Writing – review & editing. EB-R: Writing – review & editing. GP-R: Writing – review & editing. XG-P: Writing – original draft, Writing – review & editing. AT: Writing – original draft, Writing – review & editing.
